# Ian Bruce Sneddon (1915–1987)

**DOI:** 10.1007/s00415-015-7997-8

**Published:** 2016-01-02

**Authors:** Andrzej Grzybowski, Jarosław Sak, Joanna Żołnierz

**Affiliations:** Department of Ophthalmology, Poznań City Hospital, ul. Szwajcarska 3, 61-285 Poznan, Poland; University of Warmia and Mazury, Olsztyn, Poland; Department of Ethics and Human Philosophy, Medical University of Lublin, ul. Staszica 4/6,102 (Collegium Maximum), 20-059 Lublin, Poland

**Fig. 1:**
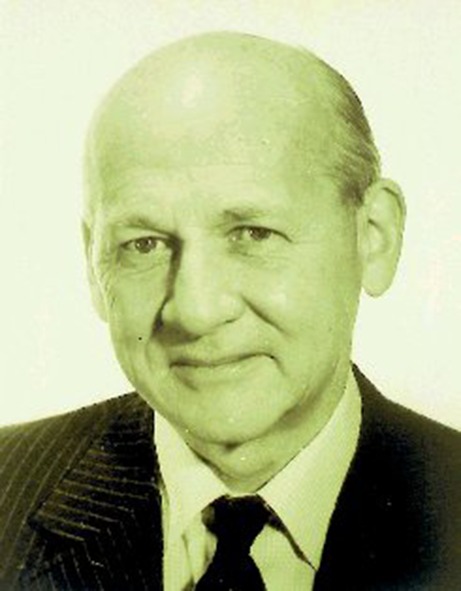
Ian Bruce Sneddon (1915–1987). http://topicalsteroidaddiction.weebly.com/chapter-2312288drsneddonrsquos-message.html

The year 2015 marked the hundredth anniversary of the birth of Ian Bruce Sneddon (Fig. 1). He was a dermatologist who contributed to the development of neurology by describing a form of non-inflammatory arterio-occlusive disorder which may manifest with stroke or other severe central nervous symptoms, and a livedo racemosa of the skin (Sneddon syndrome) [1, 2]. He was also one of the pioneers of modern psychodermatology [3] and contributed to the development of the knowledge about the nutritional neuropathy [4].

Ian Bruce Sneddon was born on March 6, 1915, in Sheffield in England. He was the only son of Kathleen Hield and Scottish general practitioner William Sneddon [5, 6]. Ian attended Uppingham School in Rutland, a school with tradition reaching back to 1584. Next, he studied at the University of Sheffield, where, in 1937 he graduated from medical studies.

Significant for the direction of his future work was his first medical practice. Sneddon worked with outstanding dermatologist Arthur Rupert Hallam (1878–1955) [7] at Sheffield Royal Infirmary who encouraged him into the specialty [6, 8]. In 1938, Sneddon became a clinical assistant in the skin department. During the Second World War, he served in the Royal Naval Volunteer Reserve as a skin and medical specialist [8]. He spent 2 years at sea, in the Pacific region, some of that time in Australia in Sydney Harbor, where his love of sailing was started. Often he sailed together with the British physician and geneticist Cyril Astley Clarke (1907–2000), later knighted and President of the Royal College of Physicians (1972–1977).

After demobilization in 1946 Sneddon, having reached the rank of surgeon lieutenant commander, returned to Sheffield and obtained the post in the skin department at the Royal Infirmary as a supernumerary registrar [5, 6, 8]. In the same year, Sneddon married Joan Simon who was a psychiatrist. They had three daughters and two sons. Sneddon and his wife shared interests and together studied the psychiatric aspects of skin disease [6].

In 1950, he became Clinical Dean and held this appointment for the next 18 years. He contributed to changes in the tutorial system for medical students. In line with the new program, a member of the consultant staff, who had been specially appointed to this assignment, helped to provide students with support.

Sneddon was promoted consultant physician for diseases of the skin at the Rupert Hallam Department of Dermatology, Royal Hallamshire Hospital in Sheffield [5, 6]. In 1957, Sneddon became the senior dermatologist at the Royal Infirmary. In 1974 he was awarded the CBE, a visiting Professorship at the University of Texas and in 1980 an honorary MD by Sheffield University. Sneddon was president of the British Association of Dermatologists from 1970, the section of dermatology of the Royal Society of Medicine 1980–1981, the North of England Dermatological Society and of the Sheffield Medico-Chirurgical Society. In 1968, because of health problems, he gave up the office of Clinical Dean. He continued in private practice and scientific work and devoted himself to his the greatest passions—sailing and gardening [6]. He was very proud of passing the examination for Yachtmaster’s Certificate. He died on 10 October 1987, aged 72. In his honor, the British Association of Dermatologists endowed a trophy for dinghy sailing in his name (the Bowers–Sneddon cup) [8].

During WWII, Sneddon observed many cases of a neuropathy in a group of prisoners of war repatriated from Hong Kong [8]. He noted the presence of ophthalmological and neurological disorders: impairment of visual acuity resulting from central and paracentral scotomata, partial optic atrophy, macular degeneration, failure of hearing, as well as swelling of the ankles, paresthesia of the limbs, difficulty in walking. Analyzing about 200 sick prisoners who were ferried to the Royal Naval Hospital in Sydney in September 1945, he published together with Cyril Astley Clarke a paper [4] and they produced a film on nutritional neuropathy among prisoners of war. They connected these disorders with a toxic and antivitamin principle in the diet, associated with vitamin B complex deficiency [4].

Sneddon was the author more than 100 articles, including on the psychological and psychiatric aspects of skin disease. He suggested a three level classification of the skin conditions in which psychogenic factors play a role in causation: dermatoses always psychic in origin (e.g. acarophobia or trichotillomania), dermatoses with a large psychogenic factor (e.g. neurodermatitis or atopic eczema) and dermatoses sometimes precipitated by psychogenic factors (e.g. psoriasis, urticaria or alopecia areata). Sneddon separated psychodermatoses from psychosomatic disease [3].

In a paper which was read first at the Annual Meeting of the British Association of Dermatology in 1964 and subsequently published in 1965 Sneddon reported six patients with severe neurocutaneous disorder [2]. All of them had “multiple cerebrovascular incidents of limited and benign nature” and “livedo reticularis” [2] (in English medical language term “livedo reticularis” is used for all types of livedo [9]). Although, the association between livedo racemosa (reticularis) and cerebrovascular disease had been described earlier by J. Kimming in German in 1959 [10], Sneddon’s analyses of the cases were more in-depth and conclusive [2]. The term “Sneddon’s syndrome” is used to describe a rare non-inflammatory thrombotic vasculopathy characterized by the combination of livedo racemosa with cerebrovascular disease [10].
